# Decadal patterns of disaster damage and recovery-to-damage dynamics in South Korea (2015–2024) with a context-sensitive comparison to Japan

**DOI:** 10.1371/journal.pone.0343670

**Published:** 2026-06-05

**Authors:** Kihun Nam, Jiwon Yoon, Jung Kyu Park

**Affiliations:** 1 Department of Fire and Disaster Prevention, Inje University, Gimhae–si, Gyeongsangnam–do, Republic of Korea; 2 CHARIS College of Liberal Arts, Changshin University, Changwon–si, Gyeongsangnam–do, Republic of Korea; 3 Division of AI Convergence, Major in Computer Science, Daejin University‌‌, Pocheon–si, Gyeonggi–do, Republic of Korea; Ibn Tofail University Faculty of Sciences: Universite Ibn Tofail Faculte des Sciences, MOROCCO

## Abstract

Understanding disaster recovery is essential for effective risk management, yet recovery processes are often implicitly assumed to scale proportionally with disaster damage. This study examines disaster damage and recovery patterns in South Korea and Japan to assess whether recovery dynamics are linear, homogeneous, and comparable across contexts. Using official national statistics, we analyze annual disaster damage and recovery expenditure in South Korea from 2015 to 2024, disaggregated by hazard type and administrative region, and compare these patterns with disaster-related fatalities in Japan from 2015 to 2023. For South Korea, results show substantial interannual variability in recovery expenditure, while statistical analysis indicates a strong and approximately proportional relationship between damage and recovery at the aggregate level. Hazard-specific analysis reveals variation in recovery intensity across disaster types, while regional analysis highlights notable spatial disparities. Extreme disaster years exhibit deviations from proportional scaling, suggesting that large events can trigger additional recovery processes beyond direct asset restoration. The cross-national comparison illustrates that disaster response indicators show different temporal patterns between South Korea and Japan when aligned with national disaster management priorities. While recovery-to-damage ratios capture fiscal recovery intensity in South Korea, disaster-related fatalities reflect the human impacts emphasized in Japan. Comparisons using scaled indicators further show that temporal response patterns do not peak synchronously across the two countries, underscoring structural differences in disaster management systems. Overall, the findings suggest that disaster recovery is a context-dependent process shaped by hazard characteristics, spatial conditions, and institutional priorities, with variability emerging around an underlying proportional relationship. Meaningful international comparisons therefore require indicator choices sensitive to national frameworks rather than uniform metrics. This study provides empirical evidence supporting a more nuanced approach to analyzing and comparing disaster recovery across countries.

## Introduction

Natural disasters continue to impose substantial social, economic, and human losses worldwide, and their impacts are expected to intensify under climate variability and increasing exposure of assets and populations [1–3]. As a result, understanding not only disaster damage but also post-disaster recovery processes has become a central concern in disaster risk management research. While damage assessments quantify the immediate consequences of hazard events, recovery processes determine how societies absorb shocks, restore functionality, and invest in resilience for future events [4,5].

Despite its importance, disaster recovery has often been treated implicitly as a proportional extension of damage magnitude. Many empirical studies focus primarily on estimating losses or attributing damage trends to climatic drivers, with recovery expenditure assumed to scale mechanically with reported damage [6–8]. However, growing evidence suggests that recovery is shaped by a broader set of factors, including institutional arrangements, fiscal capacity, policy priorities, and hazard-specific characteristics [9,10]. These factors can produce substantial variation in recovery outcomes even when damage levels are similar, calling into question the adequacy of damage-only perspectives [11]. Recent literature also suggests that resilience research in the context of natural hazards has expanded substantially in scope and methodological diversity, reinforcing the need for context-sensitive interpretation of disaster outcomes [12].

South Korea provides a useful empirical setting for examining these issues. The country maintains detailed official statistics on disaster damage and government-supported recovery expenditure, enabling systematic analysis of recovery dynamics over time, across hazard types, and among administrative regions [13]. Yet, comprehensive empirical assessments that integrate these multiple dimensions remain limited. In particular, few studies have examined whether recovery expenditure in South Korea scales linearly with damage, or how recovery efficiency varies across hazards and regions [14].

Beyond single-country analysis, cross-national comparison offers an additional lens for understanding disaster recovery. However, international comparisons are complicated by differences in data availability, economic structure, and disaster management priorities. Applying identical indicators across countries may obscure, rather than clarify, meaningful differences [15]. For example, while some countries emphasize fiscal recovery and infrastructure restoration, others prioritize minimizing human losses and displacement [16,17]. Comparative studies must therefore carefully select indicators that reflect national disaster management frameworks rather than imposing uniform metrics.

Japan represents a contrasting case to South Korea in this regard. As one of the world’s most disaster-prone countries, Japan has developed a disaster management system in which human impacts—particularly disaster-related fatalities—are a central performance indicator [18]. Comparing South Korea and Japan thus offers an opportunity to examine how different national priorities shape observable disaster outcomes and responses, provided that indicators are chosen in a context-sensitive manner. Recent studies have emphasized that the economic impacts of extreme weather events and the effectiveness of adaptation policies play a crucial role in shaping post-disaster recovery processes beyond direct damage estimates [19].

Motivated by these gaps, this study aims to address three interrelated questions. First, how do disaster damage and recovery expenditures evolve over time in South Korea, and do they generally follow a proportional relationship, with deviations in extreme years? Second, how do recovery dynamics vary across hazard types, regions, and extreme disaster years within South Korea? Third, how do disaster response patterns differ between South Korea and Japan when indicators aligned with national disaster management priorities are used?

To answer these questions, the study conducts a multi-layered empirical analysis using official government statistics. Section 2 describes the data sources, indicator definitions, and analytical framework. Section 3 presents results on temporal dynamics, hazard-specific and regional heterogeneity, extreme-year behavior, and cross-national comparison. Section 4 discusses the implications of these findings for understanding disaster recovery as a context-dependent process with deviations from proportional scaling, as well as methodological considerations for comparative disaster research. Section 5 concludes by summarizing key insights and outlining directions for future research.

By integrating temporal, structural, and comparative perspectives, this study contributes to the disaster risk and recovery literature by demonstrating that recovery outcomes cannot be inferred solely from damage magnitude and that meaningful international comparison requires indicators sensitive to national contexts.

## Materials and methods

### Data source‌‌

This study employs official disaster statistics from South Korea and Japan to conduct a comparative analysis of disaster impacts and response patterns. All data were obtained from publicly available government sources to ensure transparency and reproducibility [20,21].

For South Korea, disaster damage and recovery expenditure data were collected from the Annual Disaster Yearbook published by the Ministry of the Interior and Safety [20]. The dataset covers a ten-year period from 2015 to 2024 and includes annual records of total disaster damage and government-supported recovery costs. Recovery expenditure refers to government-supported recovery costs reported in the Annual Disaster Yearbook. It does not include private insurance payouts, non-governmental donations, household out-of-pocket expenditures, or loan-based recovery finance unless such amounts are recorded within the official government-supported recovery expenditure category.

In this study, total disaster damage refers to the annual aggregate of direct economic losses, including both private-sector damage and public-facility damage, as defined in the official Annual Disaster Yearbook. In addition, disaggregated statistics by disaster type and administrative region were used to examine structural variations in recovery responses.

For Japan, disaster-related fatality data were compiled from the Statistical Yearbook of Japan and supplemented with official disaster white papers for recent years [21]. Annual counts of fatalities caused by natural disasters were collected for the period from 2015 to 2023. These data represent officially reported deaths directly attributed to natural hazard events and are widely used as a key performance indicator in Japanese disaster management assessments.

### Disaster impact and response indicators

Different indicators were adopted for South Korea and Japan to reflect country-specific disaster management frameworks.

In South Korea, the primary indicator of disaster response intensity is the Recovery-to-Damage Ratio (RDR), defined as the ratio of total recovery expenditure to reported disaster damage within a given year. This indicator captures the relative scale of post-disaster financial intervention and has been widely used to assess recovery efficiency and fiscal responsiveness. Annual RDR values (Eq 1) were calculated as:


$$\begin{array}{c} RDR_{t}=\frac{Recovery_{t}}{Damage_{t}} \end{array}$$ (1)

where *Damage*_*t*_ and *Recovery*_*t*_ denote the total disaster damage and recovery expenditure in year *t*, respectively.

In contrast, Japan’s disaster management system places stronger emphasis on minimizing human losses. Accordingly, the number of disaster-related fatalities was selected as the primary indicator of disaster impact. Fatality counts provide a direct measure of human vulnerability and are less affected by cross-national differences in asset valuation, insurance coverage, and economic structure. Rather than imposing a uniform metric across countries, this study intentionally adopts different indicators to capture the core dimensions emphasized within each national disaster response system.

### Analytical framework

The analysis proceeded in three stages.

First, temporal trends in disaster damage and recovery were examined for South Korea to identify long-term patterns and interannual variability. This step provides baseline context for understanding recovery dynamics.

Second, structural heterogeneity was analyzed by disaggregating data by disaster type and administrative region. Disaster-type analysis highlights differences in recovery efficiency across hazard categories, while regional analysis reveals spatial disparities in recovery responses.

Third, a comparative analysis between South Korea and Japan was conducted using both raw and normalized indicators. Raw indicators were used to illustrate country-specific disaster response characteristics, while normalized indices were employed to support a descriptive comparison of temporal response patterns after removing differences in units and magnitude. Table 1 summarizes the indicator choices and analytical framework used for the comparative analysis between South Korea‌‌ and Japan.

**Table 1:** Comparison of disaster indicators and data frameworks between South Korea and Japan.

Category	South Korea	Japan
Primary disaster indicator	Recovery-to-damage ratio (RDR)	Disaster-related fatalities
Rationale	Captures fiscal recovery intensity	Reflects emphasis on human impact
Data source	Annual Disaster Yearbook	Statistical Yearbook of Japan
Temporal coverage	2015–2024	2015–2023
Unit of analysis	Annual national totals	Annual national totals
Policy emphasis	Post-disaster fiscal recovery	Human loss mitigation

### Visualization and statistical presentation

Results were primarily presented through descriptive visualizations, including line plots, bar charts, and scatter plots. These figures were designed to emphasize relative differences, temporal variation, and structural contrasts rather than statistical inference. Extreme values and outliers were retained in all analyses, as they represent meaningful disaster events rather than measurement errors. In particular, years with unusually high recovery expenditure or fatality counts were interpreted as indicators of large-scale or complex disaster impacts. All data processing and visualization were conducted using Python-based analytical tools. The analytical workflow was designed to be fully reproducible using the publicly available datasets cited above.

### Statistical analysis

To complement the descriptive analysis, additional statistical tests were conducted to quantitatively evaluate key relationships observed in the data.

First, the relationship between annual disaster damage and recovery expenditure in South Korea was assessed using Spearman’s rank correlation coefficient, which is robust to non-normality and small sample sizes. Second, a log–log linear regression model was employed to examine the scaling relationship between damage and recovery, expressed as:


$$\begin{array}{c} log(Recovery)=\beta 0+\beta 1\cdot log(Damage)+\varepsilon \end{array}$$ (2)

This approach enables interpretation of proportional versus non-proportional scaling behavior. Because the annual series contains only ten observations and the monetary values are reported in current–year nominal terms, the log–log regression was used as a descriptive scaling summary rather than as a causal, predictive, or time-series model.

Third, differences in recovery efficiency across disaster types were evaluated using the Kruskal–Wallis test, a non-parametric method suitable for comparing distributions across multiple groups. Finally, sensitivity analyses were performed to assess the robustness of results by excluding extreme observations, including the year 2020 (national extreme event) and the 2016 heavy snow outlier. All statistical analyses were conducted in Python (version 3.10.12) using SciPy and Statsmodels.

### Ethical considerations

This study relies exclusively on aggregated, publicly available statistical data and does not involve human subjects, personal identifiers, or confidential information. As such, ethical approval was not required.

## Results

### Annual dynamics of damage and recovery in South Korea

**Fig 1:**
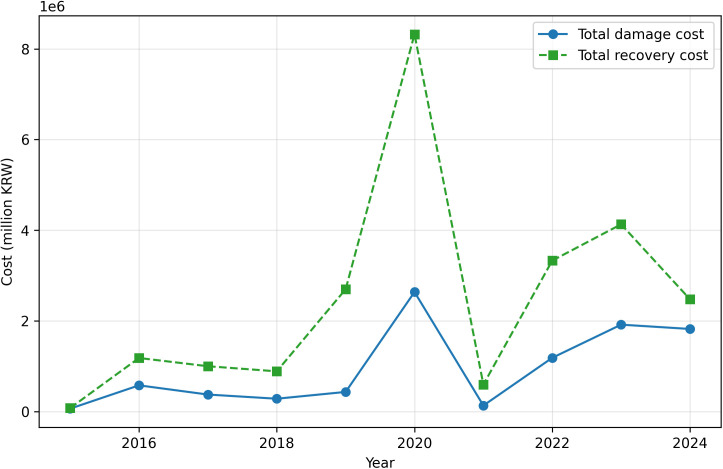
Annual total disaster damage and recovery expenditure in South Korea (2015–2024). The figure shows year-to-year variation in reported damage and government-supported recovery costs, highlighting divergence between the two series in several years.

Annual disaster damage and recovery expenditure in South Korea exhibit pronounced interannual variability over the 2015–2024 period. As illustrated in Fig 1, both series fluctuate substantially from year to year rather than following smooth long-term trends, indicating that annual fiscal pressure from disasters is strongly influenced by episodic events. Importantly, although the two series are strongly associated, several years show recovery spending that differs from a simple one-to-one correspondence with damage levels.

This visual discrepancy suggests that the recovery process is shaped not only by the magnitude of direct losses but also by additional determinants, such as recovery policy design, budgeting and implementation constraints, and the composition of affected assets. In other words, Fig 1 provides an initial empirical basis for conceptualizing disaster recovery as a distinct fiscal process rather than a mechanical consequence of damage.

**Fig 2:**
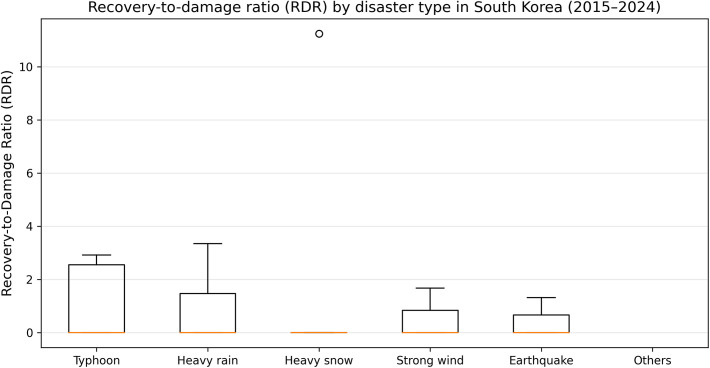
Recovery-to-damage ratio (RDR) by disaster type in South Korea (2015–2024). Boxplots show the distribution of annual RDR values by disaster type. The results illustrate descriptive variation in recovery intensity across hazards, although differences are not statistically significant.

**Fig 3:**
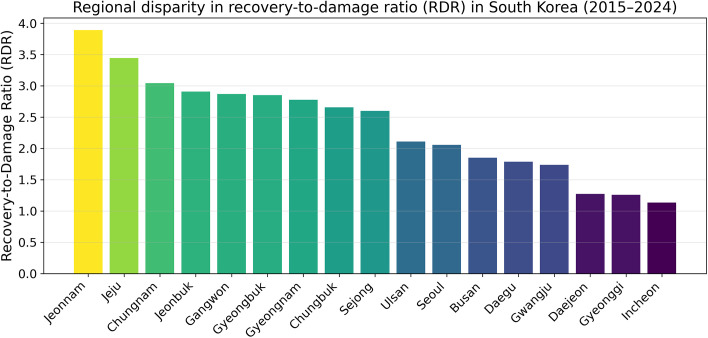
Regional disparity in recovery-to-damage ratio (RDR) in South Korea (2015–2024). This figure shows the recovery-to-damage ratio (RDR) aggregated over the 2015–2024 period for each administrative region. RDR was calculated as the ratio of cumulative recovery expenditure to cumulative reported damage. The results indicate substantial regional variation in recovery efficiency, suggesting that post-disaster recovery spending is unevenly distributed across regions.

**Fig 4:**
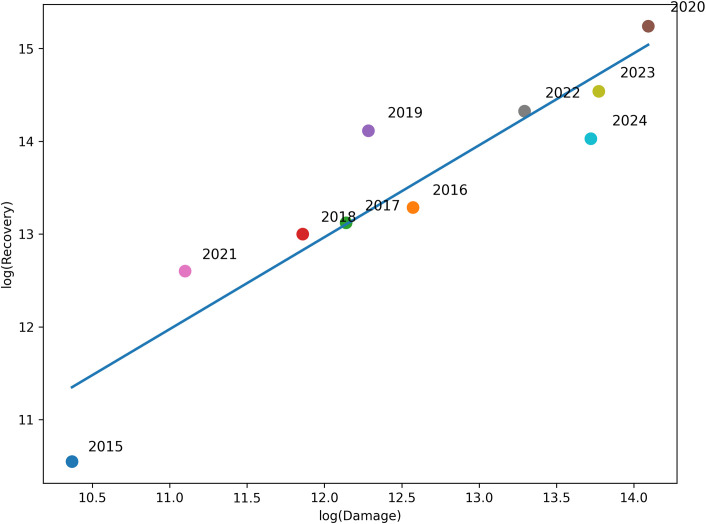
Relationship between disaster damage and recovery expenditure in South Korea. This figure plots annual total disaster damage against total recovery expenditure on a logarithmic scale. The fitted regression indicates an approximately proportional relationship, with deviations observed in extreme years.

Table 2 provides a quantitative summary of annual disaster damage, recovery expenditure, and the resulting recovery-to-damage ratio (RDR) in South Korea over the study period. While Fig 1 illustrates the overall temporal patterns, the numerical values reported in Table 2 highlight the magnitude of interannual variation in recovery intensity. For the national annual series in Table 2, the RDR ranged from 1.20 to 6.24, indicating substantial variation in annual recovery intensity relative to reported damage. Notably, even years with comparable levels of total damage exhibited different recovery intensities, indicating that RDR varies around the overall aggregate damage–recovery relationship rather than providing a simple one-to-one interpretation of recovery expenditure. For example, 2019 produced a relatively high RDR (6.24) despite lower total damage than 2020 and 2022. Because the official annual data do not provide event-level budget breakdowns, this value should be interpreted as an elevated annual recovery-intensity ratio rather than evidence of a specific fiscal mechanism. It may reflect the timing, composition, or accounting structure of government-supported recovery projects within the annual reporting framework. This quantitative baseline motivates the subsequent analyses by hazard type (Fig 2), region (Fig 3), and extreme-year behavior (Fig 4).

**Table 2:** Annual disaster damage, recovery cost, and recovery efficiency (RDR) in South Korea (2015–2024).

Year	Total damage	Total recovery cost	RDR
2015	63,724	76,245	1.20
2016	577,725	1,181,215	2.04
2017	374,605	999,344	2.67
2018	282,568	886,540	3.14
2019	432,452	2,697,519	6.24
2020	2,636,355	8,323,095	3.16
2021	132,107	594,644	4.50
2022	1,185,312	3,329,621	2.81
2023	1,916,442	4,129,980	2.16
2024	1,821,426	2,475,807	1.36

Damage and recovery costs are reported in million KRW and represent current-year nominal values (i.e., they are not inflation-adjusted) as provided in the official Annual Disaster Yearbook. The recovery-to-damage ratio (RDR) is defined as the total recovery cost divided by the total damage in each year. Total damage includes both private-sector and public-facility losses. All statistics are derived from the Annual Disaster Yearbook of South Korea.

From an interpretive standpoint, Fig 1 motivates the use of ratio-based indicators (e.g., recovery-to-damage ratio) and disaggregated analyses by hazard types and regions in subsequent figures. The observed year-to-year divergence between damage and recovery provides a direct rationale for examining where (regions) and under what circumstances (hazard types, extreme years) recovery spending deviates most strongly from damage.

### Statistical support for the damage–recovery relationship

To quantitatively assess the relationship between disaster damage and recovery expenditure, additional statistical analyses were conducted. Spearman’s rank correlation revealed a strong positive association between annual damage and recovery (ρ=0.939, *p* < 0.001), indicating that higher damage levels are consistently associated with increased recovery expenditure.

A log–log regression analysis further showed that recovery expenditure scales approximately proportionally with damage. The estimated coefficient for log(damage) was 0.991 (95% CI: 0.656–1.327, *p* < 0.001), with a descriptive model fit of *R*^2^ = 0.853. Because the confidence interval includes 1, the result supports near-proportional aggregate scaling rather than a statistically distinct departure from proportionality. Given the small annual sample size (*N* = 10), the use of current–year nominal values, and the absence of explicit time-series modeling, this regression is interpreted descriptively rather than as an inferential scaling law.

To evaluate robustness, a sensitivity analysis excluding the extreme year 2020 was performed. The relationship remained strong (ρ=0.917, *p* < 0.001), and the regression coefficient (β=0.958) showed only minor variation, confirming that the observed pattern is not driven by a single outlier year.

### Hazard-specific recovery efficiency and variability

Recovery efficiency in South Korea exhibits observable variation across disaster types. Fig 2 presents the distribution of the recovery-to-damage ratio (RDR) by hazard category, illustrating differences in both typical recovery intensity and variability. Each boxplot summarizes annual RDR values for a given hazard category, enabling comparison of central tendency (median) and dispersion (interquartile range and tails). While the figure suggests differences in recovery efficiency across hazards, these patterns should be interpreted descriptively. The observed variability indicates that recovery expenditure does not follow a strictly uniform pattern across disaster types, although the extent of these differences varies across years.

A key feature of Fig 2 is the presence of a prominent outlier in the Heavy snow category. This outlier corresponds to 2016, when recovery expenditures substantially exceeded reported damage for heavy snow events, producing an unusually large RDR value. This pattern is not treated as an error because it is consistent with the structural nature of snow-related disasters: expenditures often include response-oriented and preventive components (e.g., snow removal operations, emergency road safety measures, and infrastructure protection), which can dominate measured asset losses in the damage accounts. Consequently, the outlier provides meaningful evidence that certain hazards can generate recovery spending profiles that are qualitatively different from those implied by direct damage valuation alone.

Overall, Fig 2 supports two results. First, hazard categories exhibit observable variation in typical recovery intensity relative to damage. Second, hazard categories also differ in variability, implying that uncertainty in recovery outcomes is hazard-dependent. These findings justify hazard-aware interpretation of recovery statistics and motivate subsequent analyses examining spatial disparity and extreme-year behavior.

The effective numbers of valid annual RDR observations were Heavy rain (*n* = 10), Heavy snow (*n* = 5), Typhoon (*n* = 9), Strong wind (*n* = 3), Earthquake (*n* = 3), and Others (*n* = 0). Because the Others category contained no valid RDR values, it was excluded from the Kruskal–Wallis test. To statistically evaluate whether recovery efficiency differs across hazard types, a Kruskal–Wallis test was conducted. The result did not indicate a statistically significant difference among hazard categories (*H* = 0.166, *p* = 0.997). A sensitivity analysis excluding the 2016 heavy snow outlier yielded consistent results (*H* = 1.772, *p* = 0.778), suggesting that the observed variability should be interpreted descriptively rather than as strong statistical separation.

### Regional disparity in cumulative recovery intensity

Cumulative recovery intensity differs substantially across South Korean administrative regions. Fig 3 compares regional recovery-to-damage ratios aggregated over the 2015–2024 period, highlighting pronounced spatial disparities. For each region, total recovery expenditure and total reported damage were aggregated over the full period, and the regional RDR was computed as the ratio of cumulative recovery to cumulative damage. Because RDR is a ratio, regional values can be sensitive to small cumulative damage denominators. Therefore, high regional RDR values should not be interpreted as direct rankings of fiscal efficiency or recovery performance. The bar chart highlights substantial spatial disparities, indicating that regions differ meaningfully in the extent to which recovery expenditures are concentrated relative to reported losses.

This regional pattern suggests that recovery outcomes are shaped by factors beyond hazard exposure alone. For example, differences may reflect variation in the composition of damaged assets (public infrastructure versus private property), heterogeneous administrative capacity to execute recovery projects, and local policy priorities. Fig 3 does not attribute causality but clearly demonstrates that the recovery–damage relationship is spatially uneven at the administrative level, reinforcing the need to interpret national aggregates (Fig 1) in light of regional heterogeneity.

The magnitude of regional dispersion in Fig 3 implies that an average recovery-to-damage relationship at the national level can mask substantial local differences. As a result, policy conclusions drawn from national totals alone may be incomplete if regional conditions and recovery implementation contexts are not considered.

### Deviations from proportional scaling in extreme disaster years

The relationship between disaster damage and recovery expenditure changes noticeably in extreme disaster years. Fig 4 examines this relationship using a log–log scatter plot, allowing simultaneous visualization of typical and extreme years. Each point represents one year between 2015 and 2024, with total damage on the x-axis and total recovery expenditure on the y-axis. The logarithmic scaling is essential because it allows simultaneous visualization of typical years and extreme years without compressing the variability among smaller events.

The overall cloud of points indicates a strong positive association between damage and recovery: years with higher damage tend to require higher recovery spending. Fig 4 shows that this relationship follows an approximately proportional scaling pattern on a log–log scale. While some deviations are observed, particularly in high-damage years, these represent variations around an overall near-linear relationship rather than systematic non-linear amplification. Such deviations may reflect additional recovery components not fully captured by direct damage valuation, including public infrastructure restoration, reinforcement investments, and multi-stage recovery programs.

By highlighting and labeling extreme years, Fig 4 provides an interpretable visualization of how the damage–recovery relationship changes under large shocks. This result complements Fig 1 by showing that divergence between damage and recovery is not limited to hazard categories (Fig 2) or regions (Fig 3) but also emerges systematically at the extreme end of annual disaster severity.

### Cross-national comparison of disaster indicators

**Fig 5:**
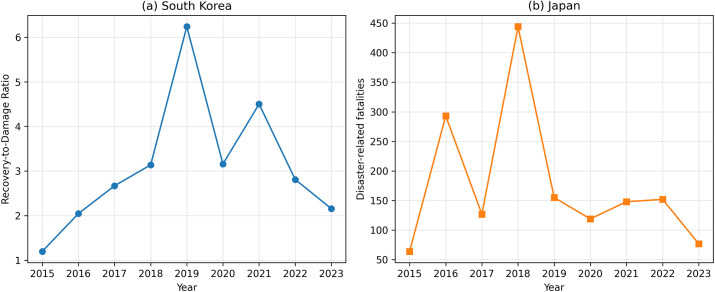
Annual disaster impact and response indicators in South Korea and Japan. **(a)** Annual recovery-to-damage ratio (RDR) in South Korea from 2015 to 2023, representing the intensity of post-disaster financial recovery relative to reported damage. **(b)** Annual disaster-related fatalities in Japan over the same period, reflecting human losses associated with natural disasters. Different indicators are used to capture country-specific disaster management priorities: financial recovery capacity in South Korea and human impact mitigation in Japan.

Cross-national comparison requires indicators that reflect each country’s disaster management priorities. Fig 5 presents a Korea–Japan comparison using country-specific indicators aligned with national frameworks. Panel (a) presents South Korea’s annual RDR over 2015–2023, capturing the intensity of post-disaster fiscal recovery relative to reported damage. Panel (b) presents Japan’s annual disaster-related fatalities for the same period, reflecting the human impact of natural disasters.

The two-panel design intentionally does not impose a single shared metric. Instead, it emphasizes that the two systems are monitored and evaluated through different core indicators. South Korea’s RDR-based pattern highlights how fiscal recovery effort varies from year to year relative to damage levels. Japan’s fatality series demonstrates that human losses exhibit strong interannual variation, with certain years standing out as particularly severe in terms of mortality burden. Together, the panels illustrate that disaster severity and societal impact can manifest through different dimensions across countries: fiscal recovery intensity on one hand, and human loss on the other.

**Fig 6:**
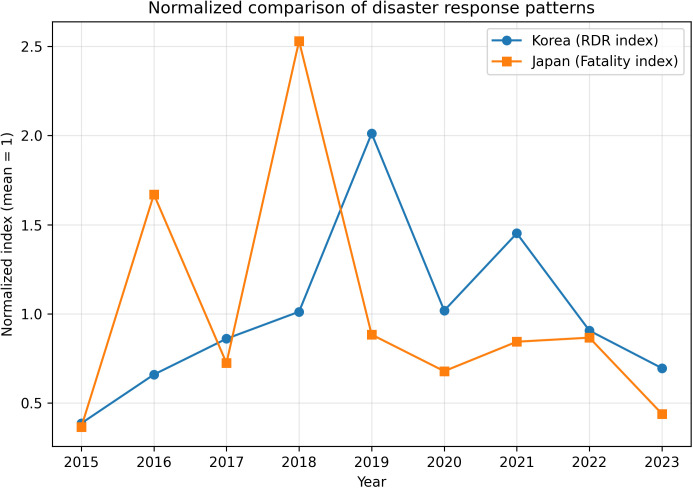
Normalized comparison of disaster response patterns between South Korea and Japan. This figure compares normalized disaster response indicators for South Korea and Japan between 2015 and 2023. Each indicator is scaled by its country-specific mean value (mean = 1) to remove differences in units and magnitude. The normalized trends highlight contrasting temporal response patterns, indicating that disaster impacts and responses vary not only in scale but also in structure between the two countries.

From the perspective of interpretation, Fig 5 establishes that a meaningful Korea–Japan comparison is possible when indicators are selected to reflect each country’s policy and statistical emphasis. It also motivates the normalized comparison in Fig 6, which removes unit differences to focus on temporal patterns.

### Normalized comparison of temporal response patterns

Even after accounting for differences in scale and units, disaster response patterns differ between South Korea and Japan. Fig 6 compares normalized indicators (*mean* = 1) to isolate temporal variability independent of magnitude. South Korea’s series is constructed by dividing annual RDR by its Korea-specific mean, while Japan’s series is constructed by dividing annual fatalities by its Japan-specific mean.

Mean normalization was used to express each annual value relative to the country-specific average over the common comparison period. This approach facilitates comparison of above–average and below-average years within each country, but it does not make the underlying indicators directly equivalent. Because mean-based scaling can be influenced by extreme years, Fig 6 is interpreted descriptively as a comparison of temporal response patterns rather than as a statistical comparison of equivalent outcome distributions. This normalization converts both indicators into dimensionless indices, allowing a descriptive comparison of within-country temporal variability without treating the underlying indicators as equivalent.

The normalized trajectories reveal that the timing and intensity of above-average years differ between the two countries. In some years, South Korea exhibits relatively elevated recovery intensity (index > 1) while Japan’s fatality index remains closer to or below its average, and vice versa. This pattern indicates that disaster impact and response do not necessarily peak simultaneously across the two countries, even within the same calendar years. The figure therefore supports the interpretation that the two national systems experience and respond to disasters with distinct temporal structures, shaped by differing hazard environments, exposure patterns, and governance mechanisms.

In sum, Fig 6 provides descriptive evidence, independent of unit scales, that the country-specific indicators exhibit different year-to-year response signatures. When combined with Figs 1–4 (within-country heterogeneity in South Korea) and Fig 5 (country-specific outcome dimensions), the results collectively support the view that disaster recovery and impact are multi-dimensional and context-dependent processes.

Taken together, Figs 1–6 indicate that disaster recovery cannot be inferred solely from damage magnitudes and that recovery dynamics show context-dependent variation across years, hazard types, regions, and extreme cases. Furthermore, the Korea–Japan comparison illustrates that national disaster systems emphasize different outcome dimensions, requiring indicator choices that respect each country’s statistical and policy frameworks.

## Discussion

### Recovery as a distinct process beyond damage magnitude

The statistical results indicate an approximately proportional aggregate relationship between disaster damage and recovery expenditure in South Korea. However, this aggregate pattern does not fully capture year-specific deviations, hazard-specific variability, or regional differences in recovery intensity.

Accordingly, the results do not indicate a broad departure from proportionality at the aggregate level; rather, they suggest that proportional aggregate scaling coexists with context-dependent deviations. Even when total damage levels are similar, recovery intensity can differ substantially, suggesting that recovery decisions are influenced by institutional, fiscal, and policy considerations beyond physical loss valuation. This interpretation is consistent with recent disaster risk management research emphasizing that state, institutional, and organizational capacities shape post-disaster response and recovery performance [22].

This finding aligns with the view that post-disaster recovery is a multi-dimensional process that includes not only asset restoration but also preventive investment and administrative implementation. Such complexity in recovery measurement has been emphasized in recent resilience literature, which notes that recovery potential cannot be inferred solely from physical damage estimates and requires context-aware indicators [11,23]. Recent recovery-planning and monitoring studies further suggest that post-disaster recovery should be understood as a system-level process shaped by infrastructure interdependencies, governance, and residents’ needs, rather than as a direct function of physical damage alone [24,25]. The presence of deviations from proportional scaling in extreme years (Fig 4) supports an interpretation that large disasters trigger recovery components beyond direct loss accounting.

### Hazard-specific and spatial heterogeneity in recovery outcomes

The hazard-specific analysis (Fig 2) highlights that recovery efficiency exhibits observable variation across disaster types. In particular, the large dispersion and extreme outliers observed for certain hazards underscore that recovery expenditure is strongly influenced by the nature of the hazard itself. For example, snow-related disasters can generate recovery costs dominated by response-oriented and preventive activities, resulting in recovery intensities that exceed those implied by direct damage estimates.

Similarly, the regional analysis (Fig 3) reveals pronounced spatial disparities in cumulative recovery intensity. These disparities suggest that recovery outcomes depend not only on exposure to hazards but also on regional characteristics such as administrative capacity, infrastructure composition, and local implementation contexts. This interpretation is broadly consistent with recent global empirical evidence showing that realized resilience after disaster events varies substantially across communities depending on local resilience conditions and recovery contexts [26], as well as with recent post-disaster recovery research showing that recovery performance depends on interactions among infrastructure systems, institutional coordination, and residents’ needs rather than on physical restoration alone [25]. Together, these results caution against interpreting national averages as representative of recovery dynamics at finer spatial or hazard-specific scales.

From a systems perspective, post-disaster recovery can be understood as an adaptive process shaped by institutional design and operational coordination rather than a purely reactive response to damage, as highlighted in recent studies employing system-level recovery frameworks [24].

### Implications of extreme years for disaster recovery planning

Extreme disaster years play a disproportionate role in shaping aggregate recovery patterns. As shown in Fig 4, years with exceptionally high damage tend to deviate from proportional damage–recovery relationships, indicating that annual recovery expenditures may depart from average-year expectations under large shocks. This finding has important implications for disaster planning, as it suggests that recovery mechanisms must be designed to accommodate increases in recovery demand that may exceed proportional expectations rather than relying on average-year assumptions. This interpretation is also supported by recent comparative evidence from 100 natural disasters, which suggests that recovery performance reflects a balance between the speed and quality of recovery and that large disasters make this balance more difficult to achieve [27].

The identification of extreme–year behavior provides an empirical basis for examining how annual recovery expenditures depart from average-year expectations. Because the present data do not directly measure institutional stress or fiscal capacity, these findings should be interpreted as evidence of expenditure variability rather than as direct evidence of recovery-system strain. Event-level fiscal data would be needed to evaluate how specific institutions or budgetary mechanisms respond to large-scale disasters.

### Cross-national contrasts in disaster response priorities

The comparison between South Korea and Japan (Fig 5) illustrates how national disaster management systems emphasize different outcome dimensions. Previous studies have shown that cross-national disaster comparisons can be misleading when uniform indicators are imposed without accounting for differences in loss reporting systems, policy priorities, and institutional contexts [15–17].

Importantly, the normalized comparison in Fig 6 demonstrates that even after removing unit and scale differences, the temporal patterns of disaster impact and response differ between the two countries. Peaks in recovery intensity in South Korea do not consistently coincide with peaks in fatalities in Japan, suggesting that the two systems experience and respond to disasters in structurally different ways. This result reinforces the argument that cross-national disaster comparisons must respect country-specific indicators to avoid misleading conclusions.

Nevertheless, the Korea–Japan comparison remains inherently asymmetric because South Korea is represented by a fiscal recovery ratio, whereas Japan is represented by a human-impact indicator. The normalized comparison therefore should not be interpreted as a direct comparison of national disaster-management performance. Rather, it provides a descriptive comparison of how country-specific disaster indicators vary over time within each national statistical and policy framework.

### Methodological considerations and limitations

Several limitations should be acknowledged. First, the analysis relies on aggregated annual statistics, which may obscure short-term dynamics within individual disaster events. Second, South Korean monetary values were analyzed as current-year nominal values, following the official reporting format of the Annual Disaster Yearbook. Because no GDP deflator or CPI adjustment was applied, the regression results should be interpreted as descriptive relationships among officially reported values rather than as inflation-adjusted estimates of real fiscal recovery intensity. In addition, the annual sample size is limited to ten observations, and the analysis does not explicitly model temporal autocorrelation or fiscal spillover across budget years.

Third, the use of different indicators for South Korea and Japan, while conceptually justified, limits direct numerical comparability and shifts the focus toward comparative patterns rather than absolute performance. Fourth, the Japanese fatality data were available up to 2023, whereas South Korean recovery data extended through 2024; although this discrepancy does not affect the main comparative findings, it should be considered in future updates. Fifth, regional RDR values may be affected by small-denominator bias, because regions with relatively low cumulative damage can produce inflated recovery-to-damage ratios. Accordingly, the regional analysis should be interpreted as a descriptive indicator of spatial variation rather than as a causal ranking of recovery efficiency.

The cross-national comparison is further limited by the absence of harmonized parallel indicators for both countries. Because RDR and disaster-related fatalities represent different dimensions of disaster response and impact, the comparative results should be interpreted as context-sensitive temporal patterns rather than as direct cross-country performance measures.

Despite these limitations, the use of official government statistics and transparent indicator definitions strengthens the robustness of the analysis. The methodological choice to retain extreme values and outliers further ensures that the results reflect real disaster experiences rather than statistical smoothing.

### Concluding implications

From an integrative perspective, the findings suggest that disaster recovery should be conceptualized as a context-dependent process shaped by hazard characteristics, spatial conditions, and institutional priorities. The South Korea–Japan comparison highlights that meaningful international comparisons do not require identical indicators, but rather indicators that accurately reflect national disaster management frameworks. By integrating temporal, hazard-specific, regional, and cross-national perspectives, this study provides a comprehensive empirical foundation for understanding disaster recovery as a complex and context-dependent process with deviations from proportional scaling.

## Conclusion

This study analyzed disaster damage and recovery patterns in South Korea and Japan using official national statistics to examine how disaster impacts and responses vary across time, hazard types, regions, and national contexts. The results show that annual recovery expenditure in South Korea is strongly associated with disaster damage and follows an approximately proportional aggregate relationship, while deviations in specific years and subnational contexts require careful interpretation.

For South Korea, recovery expenditure showed substantial interannual variability while generally following an approximately proportional relationship with reported damage. Hazard-specific and regional analyses revealed variability in recovery intensity, while extreme disaster years exhibited deviations from proportional scaling. These findings indicate that recovery processes may involve additional preventive and response-oriented components beyond direct asset restoration.

The cross-national comparison further highlights structural differences in disaster management priorities. South Korea’s disaster response is effectively characterized by fiscal recovery indicators, whereas Japan’s disaster outcomes are more appropriately reflected by disaster-related fatalities. Normalized comparisons revealed distinct temporal response patterns between the two countries, underscoring that disaster impacts and responses do not peak synchronously across national systems.

By integrating temporal, hazard-specific, regional, and cross-national perspectives, this study provides empirical evidence that disaster recovery should be interpreted through both aggregate damage–recovery relationships and the institutional contexts in which recovery expenditures are recorded and implemented. Future research may extend this approach by incorporating event-level data, inflation-adjusted fiscal series, or harmonized cross-national indicators.

## Supporting information

S1 File Annual disaster damage and recovery-to-damage ratio for South Korea. This CSV file contains annual total disaster damage, total recovery expenditure, and recovery-to-damage ratio (RDR) values for South Korea from 2015 to 2024.
(CSV)

S2 File Hazard-specific recovery-to-damage ratios for South Korea. This CSV file contains year-by-hazard RDR values used to generate Fig 2.
(CSV)

S3 File Region-level recovery-to-damage ratios for South Korea. This CSV file contains region-level cumulative disaster damage, cumulative recovery expenditure, and RDR values used to generate Fig 3.
(CSV)

S4 File Annual disaster-related fatalities in Japan. This CSV file contains annual disaster-related fatalities for Japan from 2015 to 2023 and was used in Figs 5 and 6.
(CSV)

S5 File South Korea–Japan comparison data. This CSV file combines South Korea RDR values, Japan disaster-related fatalities, and normalized values used for Figs 5 and 6.
(CSV)

S6 File Summary of reported statistical test outputs. This CSV file includes Spearman correlation, regression, sensitivity analysis, and Kruskal–Wallis test outputs.
(CSV)

S7 File Script for reproducing the statistical analyses. This Python script contains the code used to reproduce the main statistical analyses reported in the manuscript.
(PY)
